# Role of vitamin D supplement adjunct to topical benzoyl peroxide in acne: a randomized double-blinded controlled study

**DOI:** 10.1097/JW9.0000000000000163

**Published:** 2024-07-01

**Authors:** Tin Ruikchuchit, Premjit Juntongjin

**Affiliations:** a Division of Dermatology, Chulabhorn International College of Medicine, Thammasat University, Pathum Thani, Thailand

**Keywords:** 25-Hydroxyvitamin D, acne, treatment, vitamin D

## Abstract

**Background:**

Acne is an inflammatory condition of the pilosebaceous unit. Previous studies have established a link between acne and vitamin D deficiency and the potential effectiveness of vitamin D supplementation in treatment. However, the efficacy of vitamin D as an adjuvant treatment for acne remains unknown.

**Objective:**

To evaluate the efficacy of weekly vitamin D2 oral administration as an adjunctive treatment to standard topical care for acne.

**Methods:**

This study was a randomized, double-blind, placebo-controlled trial including subjects with mild-to-moderate acne. Topical 2.5% benzoyl peroxide was applied twice daily for 12 weeks to all subjects. Subjects were randomly allocated to receive either oral vitamin D2 40,000 IU weekly or placebo weekly during the treatment period. No additional treatment was administered during the 4-week follow-up period.

**Results:**

A total of 44 subjects were included in this study. All of them had inadequate 25(OH)D levels. Both regimens showed significant improvement in acne during the treatment period. Weekly vitamin D2 supplementation significantly prevented the relapse of inflammatory acne lesions (*P* = .048) at the follow-up visit. No adverse effects or biochemical changes were observed.

**Limitations:**

There were no subjects of severe acne vulgaris.

**Conclusion:**

Adjunctive weekly vitamin D2 supplementation to standard topical benzoyl peroxide could reduce relapses of inflammatory lesions in mild-to-moderate acne.

**What is known about this subject in regard to women and their families?**

- Topical retinoids are often included in treatment regimens for mild-to-moderate acne vulgaris. However, this is not recommended for women who plan to become pregnant.

**What is new from this article as messages for women and their families?**

- Without topical retinoids, oral vitamin D supplementation as an adjunctive treatment could reduce the relapse of inflammatory acne.

## Introduction

Acne is an inflammatory dermatological condition related to the pilosebaceous unit. According to the latest epidemiology of acne among the worldwide population,^1^ the overall prevalence of acne is 20.5%. Many studies^2–4^ showed the negative effects on psychological well-being, self-esteem, and quality of life in individuals with acne. Multiple factors, including increased sebum production, hormones, abnormal keratinization, bacterial proliferation, and inflammation, are involved in the pathogenesis of acne. *Cutibacterium acnes* stimulates cytokine secretion, such as interleukin (IL)-6 and IL-8, which eventually causes inflammation.^5^ According to the guideline of care for acne vulgaris,^6^ topical benzoyl peroxide (BPO) or topical retinoids and the topical combination of BPO and retinoids are the first line of treatment in mild and moderate acne vulgaris, respectively. However, topical retinoids often cause skin irritation during treatment initiation^7^ and it should be exercised among women planning a pregnancy.^8^

Vitamin D, a fat-soluble vitamin, not only regulates the function of calcium metabolism, which is important in musculoskeletal disease, but is also associated with nonskeletal diseases such as metabolic disease, malignancy, and immune disorders.^9^ The vitamin D receptor is found in many organs of the body, including the gastrointestinal tract, immune cells, brain, and skin.^10^ Many skin diseases, including lupus erythematosus, atopic dermatitis, hidradenitis suppurativa, acne, alopecia areata, and skin cancer, have been linked to vitamin D.^11^

A recent meta-analysis^12^ demonstrated significantly low vitamin D levels in patients with acne. There is also some evidence of an inverse association between acne severity and vitamin D levels. Lim et al.^13^ first revealed the benefits of oral vitamin D3 supplementation in decreasing inflammatory acne lesions compared to placebo. Ahmed Mohamed et al.^14^ showed an improvement in acne severity following active vitamin D administration.

Vitamin D can be obtained through dietary intake and synthesized from the skin.^9^ Vitamin D supplements are available in 2 forms: D2 (ergocalciferol) and D3 (cholecalciferol). A comparison of the effectiveness of vitamin D2 and vitamin D3 supplements is widely debated. However, a high dosage of vitamin D2 can be orally administered weekly to achieve the optimal vitamin D level, and the cost of vitamin D2 is cheaper than that of vitamin D3.

Therefore, this study aimed to determine the efficacy of weekly vitamin D2 oral administration as an adjunctive treatment to standard topical care for acne.

## Materials and methods

### Study design

This randomized, double-blinded, placebo-controlled trial was conducted between June 2021 and March 2022. The study protocol was conducted in accordance with the Declaration of Helsinki and approved by the Thammasat University Institutional Review Board (MTU-EC-OO-6-094/64). This study was registered in the Thai Clinical Trial Registry (TCTR 20210616002). The total duration of the study was a 16-week period divided into 2 parts: the first 12-week period was identified as the treatment period and the subsequent 4-week period was identified as the follow-up period. Informed consent was obtained from each participant prior to enrollment.

### Subjects

Participants aged 20 to 45 years who were diagnosed with mild-to-moderate acne vulgaris were enrolled. The exclusion criteria included pregnancy, lactation, history or clinically suspected of polycystic ovary syndrome, multiple sclerosis, systemic lupus erythema, sarcoidosis, diabetes mellitus, rheumatoid arthritis, renal failure, any type of liver disease, or inflammatory bowel disease and those who had known hypersensitivity to topical BPO or oral vitamin D. Use of topical medications (retinoids, BPO, antibiotics, corticosteroids, and dermocosmetics) on the face within the last 2 weeks, receiving systemic medications (retinoids, antibiotics, corticosteroids, and vitamin D supplement) within the last 4 weeks, and hormonal supplements within the last 3 months were also excluded.

The participants were randomly allocated to 2 groups according to computer-generated randomization. During the 12-week treatment period, all subjects were instructed to apply 2.5% BPO water base gel (Benzac AC, Galderma, Thailand) on their entire face for 15 minutes and then wash off twice daily. Additionally, the study group received 2 capsules of 20,000 IU vitamin D2/ergocalciferol (Calciferol, British Dispensary, Bangkok Thailand) weekly, while the control group received 2 capsules of a placebo, packed in an identical capsule, weekly. At the end of week 12, BPO and oral capsules were discontinued in all the participants. Throughout the 16-week study period, all participants used the facial cleanser and moisturizer (in-house preparation) twice daily. Concomitant use of makeup foundations, concealers, sunscreens, acne medications, and dermocosmetics was not allowed.

### Clinical assessments

Clinical photographs were taken using a standard digital camera (Mirrorless, PEN E-P5, Olympus, Tokyo, Japan) at baseline and every 4 weeks during the study. Global acne grading system score and acne counts, including inflammatory and noninflammatory lesions, were evaluated by a single blinded investigator. The Dermatologic Life Quality Index was assessed by each participant at baseline.

### Serum vitamin D assessment

Serum 25-hydroxyvitamin D (25(OH)D) levels were determined at baseline and the end of the 12-week treatment period. The specimens were analyzed within 24 hours of clotted blood collection at 2 to 8 °C storage. The 25(OH)D level was analyzed using an electrochemiluminescence immunoassay (Roche Cobas e801 Analyzer; Roche Diagnostics, Rotkreuz, Switzerland). Serum 25(OH)D level is classified as sufficient (30 ng/mL or higher), insufficient (21–29 ng/mL), and deficient (20 ng/mL or less) of vitamin D according to the Endocrine Society clinical practice guideline.^15^ Calcium and phosphate levels were also evaluated at baseline and at the end of the treatment.

### Statistical analysis

Sample sizes were estimated using n4Studies program. With a statistically significant level of .05 and a statistical power of 80%, 20 patients in each group were needed for a randomized controlled trial with continuous outcomes.

The independent sample *t* test, Mann–Whitney *U* test, chi-square test, and Fisher exact test were performed to analyze the demographic data. Spearman rank-order correlation analysis was used to determine the relationships between variables. To analyze the outcome, we used paired *t* test for comparison with baseline within groups, independent sample *t* test to evaluate between groups, and repeated ANOVA to compare all-time results between groups. IBM SPSS 27 Statistics, NY and STATA 14, TX were used for statistical analysis. Statistical significance was set at the *P* value <.05. When handling missing data, we used intention-to-treat analysis, and the last observation was carried forward.

## Results

### Baseline characteristics

**Fig. 1.**
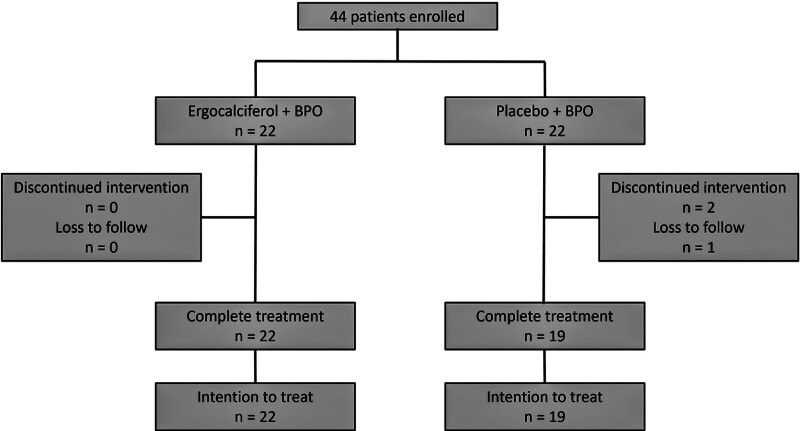
Study flow chart.

The study included 44 patients with acne. They were equally randomized into 2 groups: vitamin D and placebo. Forty-one participants (93.2%) completed the study; 3 participants from the placebo group discontinued the study because they were unable to follow-up on the COVID situation (Fig. 1). The baseline demographic information of the patients with acne is shown in Table 1. The mean age of the participants was 27 years old. Almost all participants had skin phototypes III and IV. The participants had acne onset in their teenagers with more than 10 years of experience with acne. There were no significant differences in the baseline characteristics between the experimental groups.

**Table 1:** Baseline characteristics

Characteristics	Total (*n* = 44)	Vitamin D (*n* = 22)	Placebo (*n* = 22)	*P* value
Sex, *n* (%)				.34
Male	15 (31.4)	9 (40.9)	6 (27.3)	
Female	29 (65.9)	13 (59.1)	16 (72.7)	
Age (yr), mean ± SD	27.6 ± 5.1	28.7 ± 5.2	26.5 ± 4.8	.14
BMI (kg/m^2^), mean ± SD	22.3 ± 4.2	23.1 ± 4.3	21.51 ± 4.1	.22
Fitzpatrick skin type, *n* (%)				.53
Type III	25 (34.1)	9 (40.9)	6 (27.3)	.53
Type IV	28 (63.6)	13 (59.1)	15 (68.2)	
Type V	1 (2.3)	0 (0.0)	1 (4.5)	
Sun exposure time (hour/d), median (interquartile range)	2 (1–5)	2.5 (1–5)	2 (2–5)	.66
Sunscreen use, *n* (%)				.88
Always	10 (22.7)	4 (18.2)	6 (27.3)	
Often	6 (13.6)	3 (13.6)	3 (13.6)	
Sometimes	10 (22.7)	6 (27.3)	4 (18.2)	
No use	18 (40.9)	9 (40.9)	9 (40.9)	
Mask wearing time (hour/d), median (interquartile range)	8 (3–9.5)	8 (4–8)	8 (3–11)	.76
Smoking (yes), *n* (%)	7 (15.9)	3 (13.6)	4 (18.2)	1.0
Alcohol (yes), *n* (%)	6 (13.6)	2 (9.1)	4 (18.2)	.66
Underlying disease (yes), *n* (%)	11 (25)	6 (27.3)	5 (22.7)	.73
Allergic rhinitis	4 (18.2)	0 (0.0)	4 (18.2)	
Atopic dermatitis	1 (4.5)	0 (0.0)	1 (4.5)	
Essential hypertension	1 (4.5)	1 (4.5)	0 (0.0)	
Age of acne onset (yr), mean ± SD	16.1 ± 3.6	16.5 ± 4.1	15.7 ± 3.1	.49
Duration of acne (yr), mean ± SD	11.5 ± 7.0	12.2 ± 7.6	10.7 ± 6.4	.49
DLQI score, mean ± SD	7.3 ± 1.8	8.4 ± 6.4	6.3 ± 6.0	.27
GAGS score, mean ± SD	17.7 ± 1.3	17.3 ± 5.6	18.0 ± 3.2	.58
Acne counts, mean ± SD				
Total lesions	61.8 ± 9.4	59.3 ± 34.6	64.3 ± 30.3	.61
Inflammatory lesions	16.7 ± 3.3	15.7 ± 11.9	17.7 ± 11.3	.57
Noninflammatory lesions	45.0 ± 7.1	43.5 ± 25.8	46.5 ± 23.6	.69
25(OH)D levels (ng/mL), mean ± SD	17.38 ± 1.32	16.30 ± 3.51	18.47 ± 5.22	.12

BMI, body mass index; DLQI, Dermatologic Life Quality Index; GAGS, global acne grading system; SD, standard deviation.

### Serum 25(OH)D level and the association with acne severity at baseline

The overall mean serum 25(OH)D levels were only 17.38 ± 1.32 ng/mL (9.9–28.6). According to the Endocrine Society guidelines and definition,^15^ none of the subjects with acne in this study had sufficient vitamin D levels. Moreover, there was a low inverse correlation (*r* = −0.128) between the global acne grading system score and serum 25(OH)D level, and a very low inverse correlation (r = 0.016) between total lesion counts and serum 25(OH)D levels. Nevertheless, no statistically significant differences were observed in the correlations.

### Efficacy at the end of the 12-week treatment

**Fig. 2.**
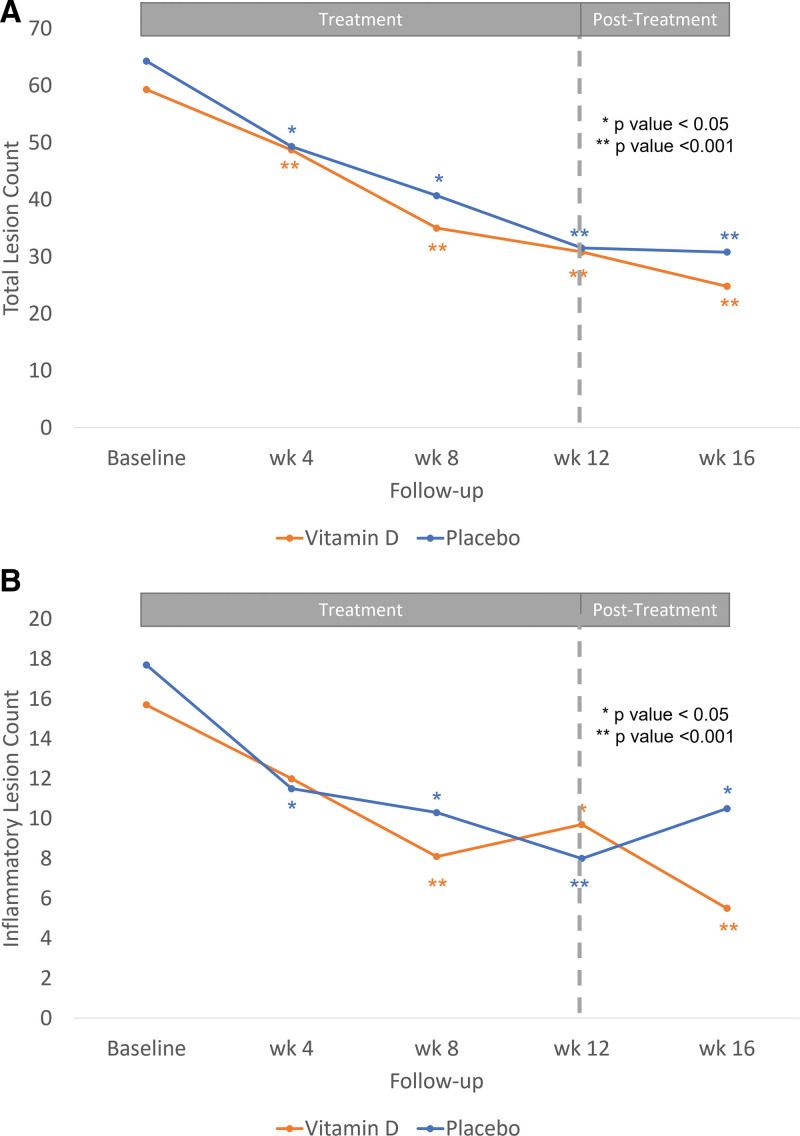
Number of acne lesion count. (A) Total lesion count. (B) Inflammatory lesion count.

At the end of the 12-week treatment, the mean total, inflammatory, and noninflammatory lesion counts of both the vitamin D and placebo groups significantly decreased from baseline (*P* < .001) (Fig. 2A and B). The total number of lesions was reduced by approximately 50% in both treatment regimens. However, there were no significant differences between the groups (Table 2). In contrast, the mean serum 25(OH)D level was significantly increased in the vitamin D group (*P* < .001).

**Table 2:** The changes of acne count lesions

Outcome	Vitamin D		Placebo		The changes between vitamin D vs placebo
	% reduction from baseline	*P* value	% reduction from baseline	*P* value	*P* value
Total lesions					
4 wk	17.87	<.001*	22.55	.004*	.898
8 wk	40.98	<.001*	36.70	.001*	.486
12 wk	48.06	<.001*	51.01	<.001*	.923
16 wk	58.18	<.001*	52.10	<.001*	.358
Inflammatory lesions					
4 wk	23.57	.101	35.03	.007*	.845
8 wk	48.41	<.001*	41.81	.005*	.510
12 wk	38.21	.017*	49.72	<.001*	.819
16 wk	64.97	<.001*	40.67	.004*	.068

* *P* < .05.

### Efficacy at the 4-week follow-up

**Fig. 3.**
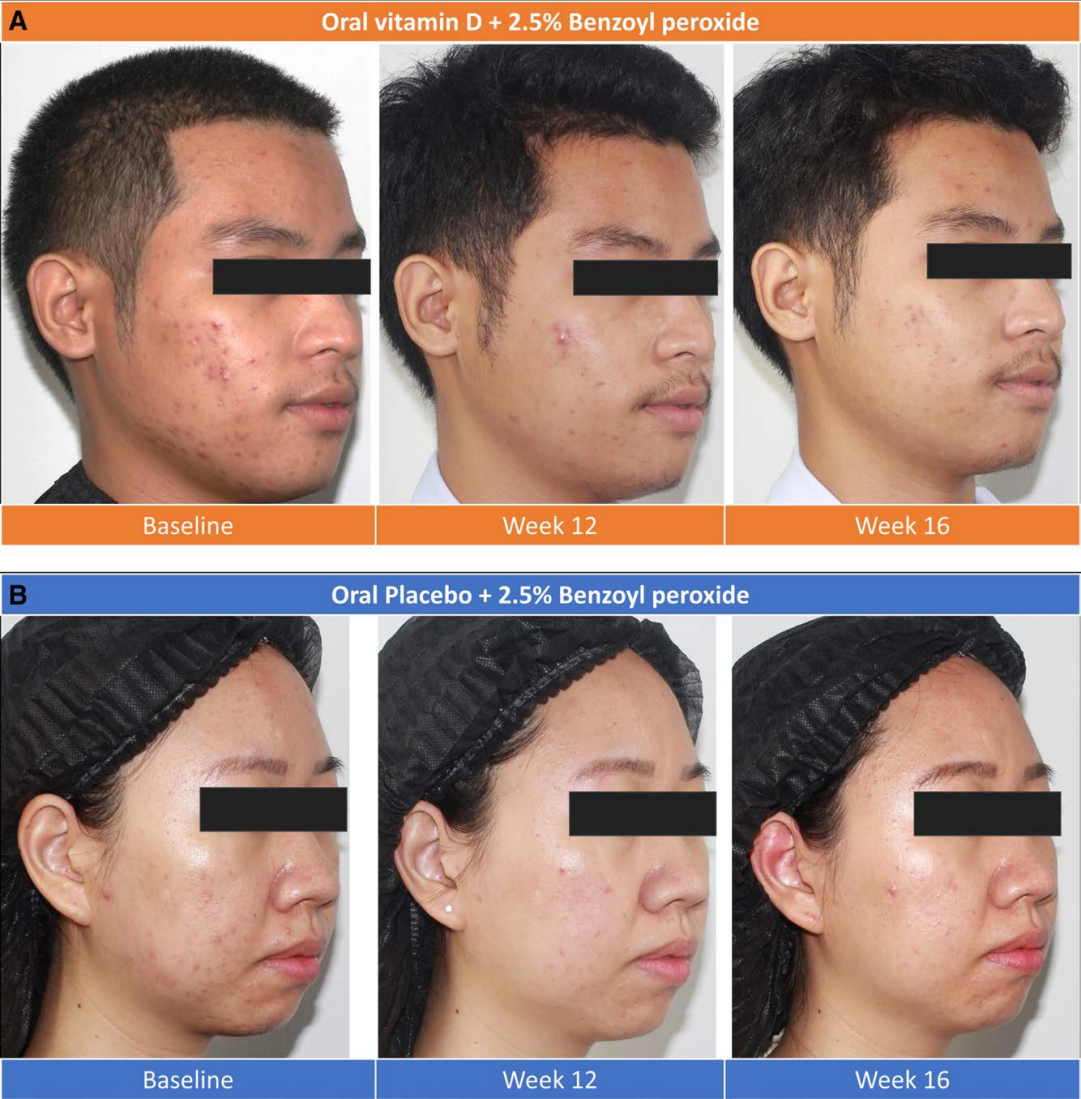
Clinical outcomes of the subjects. (A) Clinical outcomes with vitamin D supplements. (B) Clinical outcomes with placebo.

By the end of the 4-week follow-up, changes in lesion counts were calculated based on the difference between the 12th and 16th week visits. Inflammatory lesion counts were continuously reduced in the vitamin D group (approximately 38% reduction in 12th week and 65% reduction in 16th week); however, they were increased in the placebo group (from 49.72% reduction in 12th week to 40.67% reduction at 16th week). This change was a statistically significant difference between both groups (*P* = .048) (Fig. 2B). However, there were no significant differences in the changes in the total and noninflammatory lesion counts. Clinical outcomes were demonstrated (Fig. 3A and 3B).

### Serum 25(OH)D level at the end of the 12-week treatment

At baseline, vitamin D levels were insufficient in all subjects. Following the 12-week administration of 40,000 IU vitamin D2 supplementation in the vitamin D group, nearly half of the vitamin D subjects had sufficient vitamin D levels. The changes in the vitamin D status were significantly different from those in the placebo group (Table 3). There were no significant changes in calcium and phosphate levels between baseline and the end of treatment.

**Table 3:** Serum 25(OH)D levels and vitamin D status in subjects

Outcome	Vitamin D	Placebo	*P* value
25(OH)D levels (ng/mL), mean ± SD			
Baseline	16.30 ± 3.51	18.47 ± 5.22	.115
12 wk	28.67 ± 7.01	17.10 ± 4.98	<.001*
*P* value	<.001*	.333	
Vitamin D status (%)			
Baseline			.052
Insufficiency	18.2	45.5	
Deficiency	81.8	54.5	
12 wk			<.001*
Sufficiency	45.5	0.0	
Insufficiency	40.9	35.0	
Deficiency	13.6	65.0	

* *P* < 0.05.

### Safety and tolerability

Common adverse reactions from topical medications, including stinging, burning, itching sensation, peeling, and erythema, were not significantly different between the groups. Serious adverse events were not observed. There were no significant changes in the serum calcium and phosphate levels from baseline or between the groups.

## Discussion

Acne is a pilosebaceous, unit-related inflammatory dermatological disease. Noninflammatory lesions (comedones) and inflammatory lesions (papules and pustules) are often used for monitoring and severity assessment. Although several treatments are available for acne, alternative therapy or adjunctive management is needed to overcome bacterial resistance, reduce adverse effects, and enhance treatment effectiveness.

Vitamin D has been linked to cutaneous physiology, specifically, acne pathogenesis. Vitamin D can inhibit *C. acnes*-induced Th17 differentiation,^16^ lower the production of IL-6, IL-8, and metalloproteinase 9 in cultured sebocytes^17^ and promote antimicrobial peptides.^18^ Vitamin D also affects the proliferation and development of sebocytes and keratinocytes.^19^ Previous meta-analysis research^12^ demonstrated that low levels of vitamin D may be linked to acne development and potentially related to its severity. Therefore, vitamin D supplementation may be a potential adjunct treatment for acne.

Surprisingly, this study did not include a control group. Chailurkit et al.^20^ revealed that the prevalence of vitamin D deficiency in the general population of Bangkok is 64.6%. Interestingly, this study demonstrated that all the patients with acne had inadequate vitamin D levels. This may be supportive evidence of the role of vitamin D in acne pathogenesis, or it may be due to the COVID era that most people were encouraged to be indoors. A low inverse association between 25(OH)D levels and acne severity was not statistically significant. Nevertheless, we did not include patients with severe acne in this study.

After 12 weeks of treatment in this study, BPO alone and BPO in combination with weekly vitamin D2 supplements showed significant improvement in total lesion counts and inflammatory and noninflammatory lesion counts, without statistically significant differences between the 2 treatment regimens. At 16th week, a 4-week after discontinuation of all treatments, the number of total lesions steadily decreased with statistically significant differences from baseline (*P* < .001). Interestingly, inflammatory lesion counts continuously declined in BPO in combination with vitamin D supplements, whereas they tended to increase in the BPO alone regimen. The changes in the inflammatory lesions during the follow-up period between both treatments were statistically significant (*P* = .048). This finding suggests the potential role of adjunctive vitamin D as an anti-inflammatory agent in acne.

Lim et al.^13^ revealed a significant improvement in inflammatory acne following the administration of 1000 IU oral cholecalciferol (vitamin D3) daily for 8 weeks. In addition, Ahmed Mohamed et al.^14^ reported a significant reduction in inflammatory markers and improvement in acne severity after a 12-week daily administration of oral 0.25 mcg alfacalcidol. Recently, Abdel-Wahab et al.^21^ demonstrated comparable outcomes of topical calcipotriol, a vitamin D3 analog, and topical adapalene in the treatment of acne.

Therefore, any form of vitamin D supplement or topical vitamin D could be beneficial for acne treatment. The optimal dose of vitamin D supplementation to induce anti-inflammatory properties in acne remains unclear. This study proposed 40,000 IU weekly, which equates to 5,714 IU per day; this dose is within the 4000 to 10,000 IU per day range that is well tolerated and recommended by the Institute of Medicine and the Endocrine Society Guidelines.^22^

To the best of our knowledge, this is the first randomized controlled study to demonstrate the efficacy of adjunctive weekly administration of vitamin D2 in the treatment of acne vulgaris. Weekly supplements could prevent the relapse rate of acne, especially inflammatory lesions, following the discontinuation of topical medication. This regimen not only provides convenience but is also inexpensive. The limitations of this study include the lack of patients with severe acne. In addition, this study was conducted during the pandemic coronavirus; therefore, all subjects had to regularly wear masks that may conceal the real outcomes of the treatment regimens. A longer follow-up period and patients with severe acne should be included in future studies.

In conclusion, weekly vitamin D2 administration is an effective adjunctive treatment to standard topical medications to reduce the relapse of inflammatory acne.

## Conflicts of interest

None.

## Funding

Supported by Thammasat University (TUFT97/2564).

## Study approval

The study protocol was conducted in accordance with the Declaration of Helsinki and approved by the Thammasat University Institutional Review Board (MTU-EC-OO-6-094/64).

## Author contributions

TR: Participated in performance of the research, data analysis, and writing of the paper. PJ: Participated in research design, performance of the research, writing of the paper, reviewing, and critiquing it.
